# The Emerging Role of Macrophages in Chronic Obstructive Pulmonary Disease: The Potential Impact of Oxidative Stress and Extracellular Vesicle on Macrophage Polarization and Function

**DOI:** 10.3390/antiox11030464

**Published:** 2022-02-26

**Authors:** Mauro Finicelli, Filomena Anna Digilio, Umberto Galderisi, Gianfranco Peluso

**Affiliations:** 1 Research Institute on Terrestrial Ecosystems (IRET), National Research Council of Italy (CNR), Via Pietro Castellino 111, 80131 Naples, Italy; filomenaanna.digilio@cnr.it; 2 Department of Experimental Medicine, University of Campania “Luigi Vanvitelli”, Via Santa Maria di Costantinopoli 16, 80138 Naples, Italy; umberto.galderisi@unicampania.it; 3 Faculty of Medicine and Surgery, Saint Camillus International University of Health Sciences, Via di Sant’Alessandro 8, 00131 Rome, Italy

**Keywords:** COPD, macrophages, polarization, inflammation, oxidative-stress, extracellular vesicles

## Abstract

Chronic obstructive pulmonary disease (COPD) is one of the most common airway diseases, and it is considered a major global health problem. Macrophages are the most representative immune cells in the respiratory tract, given their role in surveying airways, removing cellular debris, immune surveillance, and resolving inflammation. Macrophages exert their functions by adopting phenotypical changes based on the stimuli they receive from the surrounding tissue. This plasticity is described as M1/M2 macrophage polarization, which consists of a strictly coordinated process leading to a difference in the expression of surface markers, the production of specific factors, and the execution of biological activities. This review focuses on the role played by macrophages in COPD and their implication in inflammatory and oxidative stress processes. Particular attention is on macrophage polarization, given macrophage plasticity is a key feature in COPD. We also discuss the regulatory influence of extracellular vesicles (EVs) in cell-to-cell communications. EV composition and cargo may influence many COPD-related aspects, including inflammation, tissue remodeling, and macrophage dysfunctions. These findings could be useful for better addressing the role of macrophages in the complex pathogenesis and outcomes of COPD.

## 1. Introduction

Chronic obstructive pulmonary disease (COPD) is one of the most common airway diseases, and it is characterized by progressive airflow limitation and repeated inflammation. This pathology affects patient breathing and severely limits a patient’s life and work. Given the little progress developing disease-modifying therapies that influence disease progression and mortality, more insights into the mechanisms involved in COPD development and progression are needed [1]. Besides the pivotal role played by environmental and genetic factors, inflammation and oxidative stress have emerged as key factors in COPD pathogenies [2]. 

Many different cell types are implicated in the pathogenesis of COPD. Among them, macrophages exert a key role in COPD by directly producing pro-inflammatory mediators and tissue-destructive proteases. Macrophages also accomplish this indirectly through impaired phagocytosis of bacterial pathogens and defective clearance of apoptotic cells [1]. Moreover, the link between macrophages and oxidative stress in pulmonary dysfunctions, such as asthma and COPD, is also described [3].

Interestingly, macrophages feel their microenvironment and modulate their functions through phenotype plasticity. Indeed, alteration in macrophage phenotype is a pivotal aspect for pulmonary health and respiratory disease, including COPD [4,5,6]. Based on the alterations in COPD macrophage function, it has been proposed that macrophages from COPD patients do not fit into the proposed phenotypes of macrophage differentiation [1].

Recent findings have pointed out the role of extracellular vesicles (EVs) in lung disease. EVs are lipid bilayer membrane-enclosed vesicles that arise from different lung cells, such as epithelial cells, fibroblast, and macrophages. EVs mediate intercellular communications (particularly between epithelial cells and the lung microenvironment) through their cargo, consisting of proteins, RNA, and micro-RNA, and can regulate the biology of their target cells [7,8]. 

**Figure 1:**
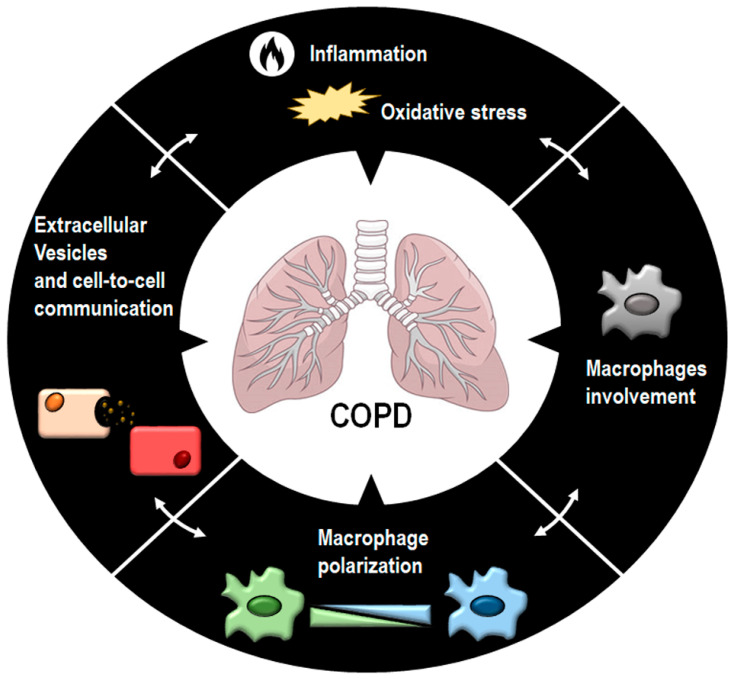
A schematic overview of the principal factors influencing COPD onset and development. COPD is a complex disease due to the concomitant action of multiple factors. In this scenario, macrophage activation and polarization, oxidative stress, and EV-driven signaling could contribute synergistically to COPD insurgency and progression.

This review focuses on the role played by macrophages in COPD and their implication in inflammatory and oxidative stress processes. Particular attention is paid to macrophage polarization, given the emerging interest in the phenotypic shifts in macrophages that could be pivotal in better understanding the role these cells play in the complex pathogenesis and outcome of COPD. We also discuss the regulatory influence of EVs in cell-to-cell communications and how they could influence the leading features of COPD, such as inflammation, tissue remodeling, and macrophage functions (Figure 1). 

## 2. COPD: Inflammation and Oxidative Stress

COPD is a common, preventable, and treatable disease. Associated with high incidence and mortality, COPD is considered a major global health problem. The issues contributing to COPD development range from genetic to environmental risk factors [9,10]. Cigarette smoking, increased exposure to particulate matter, occupational exposures, and indoor air pollution from fuel combustion are some of the features predisposing people to COPD [11]. Given the increase in air pollution, population growth, and population aging, COPD is estimated to be the main economic burden of chronic disease in the future [12,13]. It is expected to become one of the principal causes of death worldwide by 2030 [12,14].

COPD is characterized by persistent respiratory symptoms and progressively poor airflow. Its main pathological features encompass, chronic bronchitis, and pulmonary emphysema [10]. In emphysema the walls between the air sacs in the lungs are destroyed and gradually lose their shape and function [9]. This reduces the amount of gas exchange in the lungs. Chronic bronchitis is characterized by inflammation, narrowing of the small airways, and subsequent mucus production [9,15]. Although most patients with COPD show both these pathological conditions, their extent varies individually [5]. Nevertheless, the result is a progressive airway obstruction that resembles an acceleration of the normal decline in lung function due to aging [16].

The mechanisms behind COPD are still poorly understood, but chronic inflammation and oxidative stress seem to be involved in its pathogenies [16]. 

Irritant inhalation, such as cigarette smoke (CS), air pollutants, and biomass fuel smoke, activates the innate immune response. This leads to an increase in the number of neutrophils and macrophages that sustain the immune response [16]. Observations of the bronchial wall, lumen, and sputum of COPD patients have revealed an increase in the number of neutrophils. This is in line with the evidence reporting that the elastase from these cells is an important stimulator of mucus secretion [17,18,19]. Moreover, COPD patient lungs show enhanced levels of many pro-inflammatory factors, such as lipid and peptide mediators, cytokines, and chemokines [20]. The activation of these factors seems to be induced by the action of pro-inflammatory transcription factors, such as nuclear factor-κB (NF-κB) and mitogen-activated protein kinases (MAPKs) [16,21,22]. 

The lower respiratory tract of COPD patients is also susceptible to bacterial colonization. Haemophilus influenzae and Streptococcus pneumoniae colonization seem to be linked to impaired functioning of macrophages, triggering systemic inflammation and immune response. Moreover, this defect in phagocytosis may also impact the efferocytosis of apoptotic cells with a concomitant failure to resolve lung inflammation [5,16].

Besides inflammation, oxidative stress is a crucial aspect of COPD pathophysiology. Exogenous factors, such as CS and endogenous activation of inflammatory cells (i.e., neutrophils and macrophages), increase COPD patient oxidative stress levels [16]. Indeed, environmental pollutants and CS evoke an excess of reactive oxygen species and nitrogen species (ROS and RNS, respectively) [23]. In particular, ROS activate NF-κB and p38 MAPK with the consequent increase in the expression levels of pro-inflammatory genes [16]. Moreover, these molecules fuel inflammation. This leads to the production of pro-inflammatory cytokines and inflammatory cells recruitment, which generates ROS and increases the oxidative stress burden [23,24].

Oxidative stress also causes DNA damage, which is physiologically managed by the efficient DNA repair machine [16,25]. COPD patients evidence a failure in double-strand DNA breaks repair and an increase in the expression of 8-hydroxy-2-deoxyguanosine, a biomarker of DNA oxidative damage [26]. This reflects the reduction in antioxidant defenses in COPD patients due to oxidative stress status increase [27].

Based on this evidence, it is reasonable to speculate that lung macrophages may play a pivotal role in COPD pathophysiology, given their ability to regulate immune response and generate oxidants [3]. Several studies have described the increased presence of macrophages in the sputum and lungs of COPD patients, suggesting a correlation with the severity of the pathology [17,28,29,30].

## 3. Pulmonary Macrophages: Origin, Function, and Polarization 

Macrophages are specialized cells that perform a crucial role in organ and tissue innate and adaptive responses to foreign invaders and materials [17]. These cells are also involved in developmental processes and tissue homeostasis maintenance.

Macrophages are the most representative immune cells in the respiratory tract, given their role in airways surveillance, cellular debris removal, immune surveillance, and inflammation resolution [31]. Pulmonary macrophages can be divided into two populations found in anatomically distinct compartments. These are alveolar macrophages (AM), which line the alveolar surface, and interstitial macrophages (IM), which populate the space between the alveolar epithelium and vascular endothelium [6,31,32]. The expression patterns of the integrins CD11b and CD11c are commonly used to mark these cells. AMs show high expression levels of CD11c with a concomitant absence of CD11b. 

Conversely, the IM signature is characterized by high levels of CD11b and low levels of CD11c [31]. Besides their location and integrin expression levels, functional differences are the most interesting features of these cells. AMs, representing 90% of the pulmonary macrophage population, seem to be involved in host defense and are considered primary effector cells, given their phagocytic ability. They exert this role through the secretion of antimicrobials, nitric oxide (NO), tumor necrosis factor (TNF)-α, and interferon (INF)-γ [5,31]. On the other hand, IMs are less evident in phagocytosis, seem to have a regulatory role in lung tissue, and can stimulate T-cell proliferation in vitro. Moreover, these cells release cytokines involved in the adaptative immune response, such as interleukin (IL)-10 [6,31].

Concerning the origins of these populations, AMs seem to derive mainly from embryonic progenitors that colonize lungs shortly after birth. These cells live independently from the contribution of blood monocyte-derived macrophages. Nevertheless, AM depletion or damage promotes the recruitment of circulating monocytes. This becomes an important source of new macrophages, contributing to the repopulation of the AM niche and the tissue-resident AMs [33]. In contrast, less is known about IMs, which seem to derive from both yolk-sac macrophages and bone marrow-derived monocytes [34]. Recently, Gibbings et al. proposed that IMs are a heterogeneous population defined by the differential expression of cell-surface proteins such as CD206, Lyve-1, CD11c, CCR2, and MHC class II. In particular, they identified three subpopulations with the following signatures: IM1 (MHCIIlow, CD206high, IM2 (MHCII+, CD206high, IM3 MHCIIhigh, CD206lo, and CCR2+) [35]. 

Despite their specific role and origin, AMs and IMs are the lung’s first barrier against allergens, harmful substances, and/or hosts that threaten lung homeostasis. Their essential role consists of challenging these allergens without perturbing lung functionality. Therefore, macrophages adopt phenotypical changes based on the stimuli they receive from the surrounding tissue. This plasticity is described as M1/M2 macrophage polarization, which consists of a strictly coordinated process leading to a difference in the expression of surface markers, the production of specific factors, and the execution of biological activities [34,36].

The differentiation of M1 macrophages from either naïve M0 or polarized M2 macrophages is mediated by potent inducers such as INF-γ, the combination of INF-γ with the lipopolysaccharide (LPS) exposed by the exterior membrane of Gram-negative bacteria, and cytokines (e.g., granulocyte-macrophage colony-stimulating factor (GM-CSF) and TNF-α) [31,36,37]. The leading characteristics of M1 macrophages are the release of pro-inflammatory cytokines, such as TNF-α, IL-12, IL-15, IL-23, and IL-1β; generation of reactive nitrogen intermediates and ROS via inducible nitric oxide synthase (iNOS); and high antigen-presenting capacity [6,33,36] (Table 1). Accordingly, M1 macrophages show antimicrobial and cytotoxic activities and immunostimulatory effects [34,36,38].

In contrast to M1 macrophages, M2 macrophages exert their role in tuning inflammation, supporting angiogenesis, scavenging debris, and tissue remodeling.

M2 can be induced by several stimulatory factors whose exposition may, in turn, give rise to a subset of phenotypes termed M2a, M2b, and M2c (Table 1). IL-4 and IL-13 initiate the M2a subtype and induce a negative regulation of pro-inflammatory molecules (e.g., INF-γ, TNF-α, IL-6, IL-12, and IL-1β) along with superoxide anion production. Other factors regulated by these cytokines are beta 2 integrins, metalloproteinases, and the tissue-type plasminogen activator [36]. M2b is elicited by an immune complex, IL-1 receptor ligands, and LPS. M2b is characterized by a decrease in IL-12 levels and an increased production of IL-10 [34,36]. M2c is stimulated by glucocorticoids, TGF-β, or IL-10 and shows low production of pro-inflammatory cytokines [33,36]. Wang and colleagues proposed an additional subtype, M2d, triggered by the presence of tumor-associated factors and characterized by a reduced expression of IL-12 and an enhanced release of IL-10 [36,39]. 

Although alteration in macrophage phenotype is a pivotal aspect for pulmonary health, it is also linked to respiratory disease, including COPD [4,5,6]. The macrophages appear extremely dynamic during inflammatory disease, ranging from M1 to M2 phenotypes to cope with the immunological needs arising from the lung microenvironment [31].

## 4. Macrophages and Their Polarization in COPD

It has been widely acknowledged that mice are the most commonly used organisms to study human diseases. Of note, human and mice show high similarity in their immune system, both consisting of mononuclear phagocytes, granulocytes, and lymphocytes [40]. Although many findings evidenced in mice have been successfully translated for therapeutic use in human, some limitations still exist. Thus, in this review we provide a clear indication about the experimental source (human or murine) of the evidence reported. 

### 4.1. Macrophages in COPD

Macrophage increases in the sputum and lungs of COPD patients have been widely acknowledged as proof of macrophage involvement in COPD. This is sustained by cumulative findings showing the role played by macrophages in the inflammatory process and tissue destruction triggered by COPD [31,41,42]. The ability to produce most of the mediators highly expressed in the pulmonary tissue of COPD patients is one of the key speculations about the orchestrating capacity of macrophages in COPD [43]. The exposure to CS and particulate matter induces the macrophage release of molecules, driving both innate and adaptive responses. This, in turn, influences local lung inflammation, determining the development and progression of COPD [44]. In addition, macrophages produce high levels of mediators, such as IL-8 and granulocyte-macrophage stimulating growth factors (GM-CSF), which drive neutrophil recruitment and macrophage maturation [43]. Besides cytokines and chemokines, macrophages also produce and release proteases, including matrix metalloproteases (MMPs), which are considered critical in destroying the extracellular matrix (ECM) and amplifying the immune response. These events are linked to the tissue destruction observed in COPD lung tissue and are likely associated with emphysema [43,44]. Evidence from the macrophages of COPD patients compared with non-smokers revealed an imbalance of an MMP and its inhibitor. Russel and colleagues showed that macrophages obtained from COPD patients produce high levels of MMP-9 with a concomitant loss in the release of the tissue inhibitor of metalloprotease (TIMP)-1 [45]. 

Although the production of pro-inflammatory mediators and tissue-destructive proteases drive the direct contribution of macrophages to COPD, these cells play an indirect role due to the impairment of phagocytosis and efferocytosis [1]. COPD lungs are colonized by bacterial species, such as H. influenzae, *S. pneumoniae*, and Moraxella catarrhalis, despite the 20-fold increase in macrophage numbers. This suggests a defect in the bacterial clearance ability of these cells [43,46]. Other studies in human and animal models of COPD seem to confirm the defect in phagocytosis of lung macrophages [44,47,48,49]. Singh et al. used a human monocyte-derived macrophage model to assess the associations between macrophage phagocytosis and exacerbation frequency. Their results evidenced defective phagocytosis of H. influenzae by COPD macrophages associated with exacerbation frequency. Moreover, they observed a pro-inflammatory attitude of these cells, likely contributing to disease progression. The authors speculated that targeting systemic inflammation and macrophage functions during exacerbation may counteract COPD injuries [50]. 

A defect in efferocytosis, defined as the ability to remove cell debris and dead or damaged cells due to inflammatory insult, is also documented in macrophages from COPD patients [46]. In a seminal study, Hodge et al. first described a defect in phagocytosis of COPD AMs, obtained from human BAL. The authors observed that these cells could not remove apoptotic epithelial cells. On the contrary, COPD-derived cells showed no defects when polystyrene beads were used to assess phagocytosis ability. These findings suggested a failure to recognize apoptotic markers rather than a generalized phagocytic impairment [51,52]. 

Further studies confirmed this evidence [53,54,55]. Although the mechanisms are still not clear, it has been proposed that high levels of oxidative stress and proteolytic reactions may reduce the efferocytic opsonins and cleave efferocytic receptors. This could account for the increase in apoptotic cells and debris in COPD patient lungs [44]. Overall, these observations suggest that defects in phagocytosis and efferocytosis of lung macrophages could contribute to bacterial colonization and consequently expose COPD patients to exacerbation risk [46].

### 4.2. Macrophage Polarization in COPD

As previously evidenced, the microenvironment of the lungs is extremely dynamic due to repeated exposure to external microbes and environmental toxins. This leads to the need for a high modulatory capacity in the immune response that macrophages accomplish with the polarization ability. Nevertheless, the role of distinct macrophage phenotypes in COPD is still a topic of much debate. It has been widely acknowledged that the strict dichotomy of the M1/M2 classification is quite simplistic, given the wide range of macrophage phenotypes. However, this nomenclature is still used for addressing the description of macrophage response and its role in human diseases [4,6]. 

CS is one of the leading risk factors for COPD. Of note, many compounds found in cigarette extracts can induce the M1 macrophage phenotype. The increased iNOS expression in the AMs of COPD patients supports this, suggesting a polarization toward an M1 phenotype. The upregulation of iNOS sustains the ROS- and NO-induced oxidative stress, which is an essential contributor to COPD pathogenesis [6,17]. Other evidence reported that smoke exposure contributed to the release of M1 pro-inflammatory cytokines, such as IL1β, IL-6, IL-8, and TNF-α [6,56,57,58]. 

Interestingly, these molecules are elevated in COPD, and they have been associated with the development of persistent inflammation, emphysema, and mucus production in experimental settings [6,17]. These findings suggest a contribution of M1-derived cytokines in COPD pathogenesis. M1 macrophages also show increased production of MMP9 to sustain the migration of these cells during inflammatory response [59]. This protease plays a key role in COPD, as it promotes the breakdown of ECM. Indeed, macrophages from COPD patients show increased production of MMP9 compared to those from the control [6,45].

M2 macrophage dominance has been observed in the bronchoalveolar lavage (BAL) of COPD subjects sustained by producing related cytokines, such as IL-4, IL-13, and IL-10 [60]. It has also been suggested that AMs are triggered toward an M2 polarization by smoke along with the expression of MMP-12 [6,17]. MMP-12 seems to be induced by IL-4-activated M2 macrophages and plays a vital role in COPD. Its production by macrophages hampers inflammation and promotes tissue remodeling, and it seems to perform a critical role in mouse emphysema [6,61,62]. Beyers et al. reported combining evidence from COPD patients and a mouse model showing an IL-13-mediated M2 polarization along with a mucin gene expression [63]. Mucus hypersecretion is one of the leading causes of airway obstruction in COPD. Other evidence suggests that the maintenance of AMs with a skewed expression of M2 wound healing-related genes impair the hampering of inflammation with concomitant induction of detrimental remodeling of lung tissue [31]. 

Nevertheless, COPD emerges as a dysfunctional macrophage-driven pathology rather than a disease associable with a particular polarization state. Recent evidence reported a dual or a non-polarization of macrophages in COPD [4,64]. Bazzan and colleagues studied the pattern of AM polarization in a cohort of persons ranging from normal subjects to severe COPD patients. Their data showed that M1 and M2 markers were both expressed in AMs, and this dual positivity was evident with the disease progress. The authors speculated that macrophages could switch polarization and also be dually polarized. Indeed, the increase of TNF-α or IL-4 and IL-13, considered as M1 and M2 activity markers, paired with the percentage of M1- and M2-AMs. This was viewed as proof of the functional activation of both phenotypes [4]. 

On the other hand, Takiguchi et al. examined the macrophage phenotypic distribution in the BAL of COPD and non-COPD individuals. They found that 25% of macrophages were negative for both CD40 and CD143 markers, to identify M1 and M2 phenotypes, respectively. Interestingly, these double-negative macrophages were more enriched in COPD patients than non-COPD subjects. The authors also studied the gene signature of these cells, which was of a pro-inflammatory type, suggesting a disfunction in metabolism and homeostasis [64]. Again, Akata et al., phenotyped the lung macrophages according to CD40 and CD163 expression profile in HIV patients and HIV patients suffering COPD [65]. They showed that HIV+COPD patients had a higher number of non-polarized macrophages (CD40-; CD163-) with respect to HIV patients, 84.1 vs. 23.9%, respectively. Their results let authors speculate that the abundance of non-polarized macrophages expose HIV+COPD patients to an increased risk of disease progression due to an impaired phagocytic ability of this cell subset [65].

**Figure 2:**
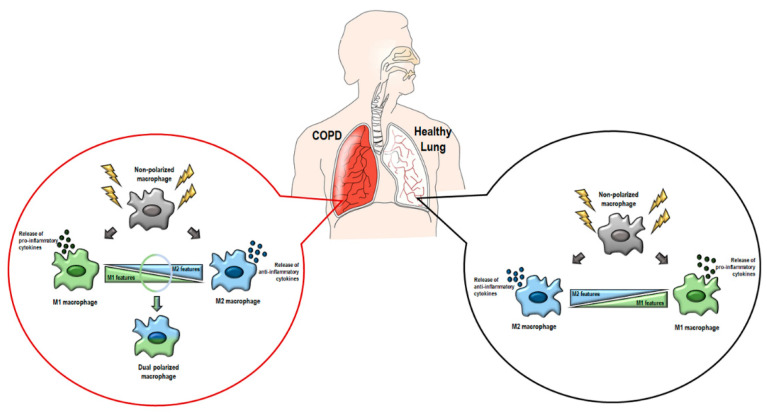
A representative illustration of the macrophage subtypes in COPD and healthy lungs. In healthy lungs (right circle), upon stimulation, macrophages could be polarized in an M1 (green) or M2 (blue) phenotype. In COPD lungs, at least four macrophage subtypes exist (left circle): non-polarized macrophages (gray), M1 macrophages (green), M2 macrophages (blue), and dual-polarized macrophages (half-green and blue).

The combination of this evidence seems to suggest that the complexity of the COPD microenvironment influences macrophage states and functions. Thus, at least four macrophage phenotypes could be identified: non-polarized macrophages (double-negative for M1 and M2 markers); M1-skewed macrophages (high expression of M1 markers); M2-skewed macrophages (increased expression of M2 markers); and hybrid macrophages (double-positive for M1 and M2 markers) (Figure 2) [46]. Interestingly, M1 macrophages seem to mainly lie in small airway wall tissues, while M2 macrophages stay in airspaces. Non-polarized macrophages are found in both compartments, and clear information about dual-positive macrophage distributions is still missing [46]. In addition, the low persistent inflammation that is causative of COPD could be induced by the coexistence of both inflammatory cytokines and immunosuppressive factors [66,67]. This engenders speculation that regulation of macrophage polarization may be a powerful tool for novel therapeutical approaches. For example, Wang et al. demonstrated the protective effect on acute lung injury evoked by manipulation of macrophage polarization [68]. They used anti-inflammatory nanoparticles in a mouse model of acute lung injury, showing their effect in alleviating tissue inflammation by increasing IL-10. These particles drove the polarization of bone marrow-derived macrophages toward an M2 phenotype in vitro and increased the M2-AM in BALF and lung tissue of mice [68]. 

These findings pave the way for in-depth knowledge about the phenotypic shifts in COPD macrophages and their biological meaning. This could be pivotal to reach a greater understanding of the role of these cells in the complex pathogenesis and outcomes of COPD. 

## 5. Oxidative Stress, Macrophage Polarization, and Phagocytosis in COPD

Oxidative stress is a key component in COPD pathogenesis, and its link with macrophages is an important feature with biological and clinical implications. ROS and RNS can negatively impact macrophage functions, thus influencing disease severity. On the other hand, macrophages are an essential source of ROS, generated in response to the host and during phagocytosis [3]. 

One of the first oxidative-stress-induced dysfunctions in macrophages is the impairment of phagocytosis. Tran et al. observed a reduction in mannose-binding lectin (MBL) in the airways of COPD patients. Given the role of this factor for the effective macrophage phagocytic functions, the authors investigated whether the oxidation of the protein could affect macrophage functionality. Thus, macrophage efferocytosis and phagocytosis of non-typable H. influenzae were measured in the presence of plasma-derived MBL before and after oxidation. The authors found that MBL oxidation was associated with reduced macrophage functionality, supporting the hypothesis that oxidative stress in the airways of COPD patients is a detrimental factor in the efferocytosis of apoptotic cells and phagocytosis of pathogens [69]. Another study on AMs isolated from the BAL of healthy donors and COPD patients showed a chronic production of mitochondrial ROS (mROS) in the latter group. Nevertheless, this increase in ROS was not sustained upon infection with *S. pneumoniae* with an impairment in bacterial clearance. These data confirm that high oxidative stress in COPD may impair mitochondrial function and metabolism-reducing phagocytosis properties in AMs [70].

Of note, it is now emerging that the changes in macrophage function and polarization during exacerbation could result from an environment characterized by high oxidative stress. Oxidative stress seems to be associated with macrophage impairment when facing pathogens due to defective phagocytosis and aberrant polarization [3].

For example, using antioxidant and anti-inflammatory natural compounds, such as propolis extract, seemed to repair lung damage due to CS in a mouse emphysema model. Interestingly, propolis treatment could induce macrophages toward an M2 phenotype with a sustained release of IL-10, thus favoring an anti-inflammatory microenvironment [71]. 

Given that many of the studies on this topic have been carried out in animal models, further research on humans is needed to prove the relevance of these findings [3]. 

## 6. Extracellular Vesicles 

### 6.1. Classification and Biogenesis

Cell-to-cell communication is an essential feature for many physiologic processes. In recent years, the role of extracellular vesicles (EVs) as an intercellular conveyance has attracted the attention of many researchers. The secretion of EVs is a conserved process from bacteria to humans and plants that was assumed to be a means of eliminating waste material from cells in the past. We now know that EVs and their content are crucial for exchanging components between cells, thus assuming profound importance in various biological systems. Indeed, these vesicles seem to play a role in communication between cells of an organism or between species [7,72]. 

**Figure 3:**
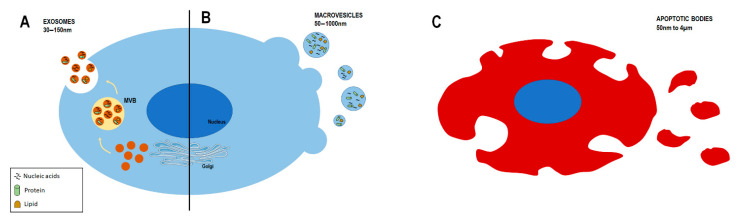
A representative scheme reporting the principal classes of extracellular vesicles (EVs) of eukaryotic cells. EVs can be divided into exosomes (**A**), macrovescicles (**B**), and apoptotic bodies (**C**), which vary according biogenetic mechanism, size, and other characteristics. MVB: multivesicular bodies.

Although the term EVs is commonly used to indicate the secreted membrane vesicles, they are a heterogeneous subset categorized according to their size and biogenesis. Three groups of EVs exist: microparticles or apoptotic bodies, macrovesicles, and exosomes (Figure 3) [73,74]. 

Apoptotic bodies are the largest EVs, ranging from 50 nm to 4 µm, and are generated during the process of apoptosis as cell-membrane blebbing and/or fragmentation [7,73,75]. Although the content of these vesicles differs depending upon cellular origin, it can consist of non-coding RNAs, DNA fragments, and cell organelles. Studies have revealed that genetic information can be transferred upon the uptake of apoptotic bodies [75,76]. 

Macrovesicles are the second group of EVs for size (50–1000 nm). They originate from the direct outward budding of the plasma membrane of living cells, and this formation is an energy-consuming process that requires ATP [73,77]. The function of macrovesicles depends on their bioactive cargo, which influences the surrounding cells through direct interaction once in the microenvironment [75]. Integrins, selectins, and CD40 are the most-used markers for identifying macrovesicles [78]. They are commonly secreted by cells in a resting state, but, interestingly, their release could also be induced upon stimulation [7].

Exosomes are the smallest EVs (30–150 nm). They are generated by early and late endosomes and form multiple vesicular bodies (MVBs) that fuse with the plasma membrane and are released in the extracellular space [2,7,73]. The formation of MVBs and intraluminal vesicles (ILVs), leading to exosome generation, is a complex mechanism that can be either dependent or independent of the endosomal-sorting complex required for transport (ESCRT) [79]. The ESCRT machinery is involved in generating polymeric fragments, ILV formation, and their release as exosomes. Indeed, proteins belonging to ESCRT components, such as Alix and tumor susceptibility gene 101 (TSG101), are present on exosomes [7,73]. On the other hand, ceramide and tetraspanin CD63 are vital components of the ESCRT-independent machinery, involved in exosome biogenesis and release [73,79]. 

The phospholipid membrane surrounding the exosome consists of cholesterol, sphingomyelin, ceramide, and lipids characteristic of the parental cell. The larger number of lipids in exosomes with respect to the cell of origin confers more rigidity to EV membranes [7,80,81]. The presence of tetraspanins, such as CD9, CD63, CD81, and CD82, is considered a marker for the exosomes. Other cytosolic proteins are present in the exosome (e.g., Rabs) and promote exosome docking and membrane fusion events. Again, annexins are also present in exosomes and are involved in regulating membrane cytoskeleton dynamics and membrane fusion events [7,82,83]. Of note, the presence of functionally active-nucleic acid cargo in exosomes has been described. This cargo consists of micro-RNA (miRNA), long non-coding RNA (lncRNA), small interfering RNA (siRNA), and RNA-protein complex [7,79,84,85]. 

### 6.2. Function

As stated before, the important function of EVs is cell-cell communications, and the current understanding sheds new light on the matter. In the last few years, EVs have emerged as a non-canonical mechanism of cytokine release in the extracellular milieu [86]. This is an important insight to understanding the mechanisms by which cytokine/chemokines achieve a proper level in the microenvironment and may reach target cells [73]. Indeed, their encapsulation in EVs could result from a defensive process the cell uses to preserve itself from the autocrine effect of these molecules or a way to safeguard the secreted cytokines from environmental degradation. The second hypothesis is more suggestive and may explain how cytokines could reach distant target cells, achieve a proper concentration at the cell surface, and penetrate cells that otherwise might not be targeted by cytokines in solution [86]. The suggestion that EVs function as carriers to concentrate and transport signaling molecules is supported by the evidence reporting the enrichment in cytokines, chemokines, proteins, and miRNAs inside these vesicles [73,87,88]. Of note, miRNAs seem to be preserved by EVs from nucleases in the extracellular milieu. Thus, the protective action of the EVs lipid bilayer could explain the stability of circulating miRNAs in body fluids. Due to exosomal miRNAs, much attention has been given to their regulatory role in many physiological functions and pathological processes [89,90].

Overall, these findings suggest the potential role of EVs for disease biomarker detection, the development of novel drug delivery systems, and the regulation of pathophysiologic processes.

## 7. Extracellular Vesicles and COPD

In normal conditions, EVs contribute to maintaining lung homeostasis via cell-to-cell communications. Lung epithelial cells are the primary source of these vesicles. Nevertheless, EV composition and secretion could be affected by external stimuli, which could, in turn, impair the influence of EVs in surrounding cells and the microenvironment [75,90]. EVs are involved in different respiratory diseases, including COPD. EVs, especially exosomes, have been demonstrated to play an essential role in the occurrence and progression of COPD. In particular, they seem to impact the regulation of fundamental processes involved in COPD, such as lung inflammation, degeneration of lung tissue, and small airway remodeling [2,91].

Concerning the contribution of EVs in airway remodeling in COPD, Fujita et al. carried out an in vitro study on human bronchial epithelial cells (HBECs) and lung fibroblast. Their data showed that CS extract (CSE) induced altered EV production. The authors identified an upregulation in cellular and EV miR-210 expression of HBEC. Of note, they demonstrated that smoke exposure modified EV components, and they identified miR-210 as a paracrine autophagy mediator of myofibroblast differentiation. They also suggested a possible implication for COPD [92]. Another study assessed the aberrant bronchial epithelium-fibroblast communication in an in vitro model. The authors used normal or CSE-treated HBECs co-cultured with bronchial fibroblast. Their data showed that CSE modified the exosome components and identified the aberrant expression of miR-21. This molecule plays a key role as a mediator of myofibroblast differentiation and contributes to airway remodeling [93]. Genschmer et al. also evidenced the role of exosomes in the pathogenesis of disorders of EMC homeostasis linked to COPD, using human in vitro and mouse in vivo models [94]. They identified activated PMN (polymorphonuclear leukocyte)-derived exosomes containing neutrophil elastase (NE) on their surface. Interestingly, these vesicles seemed to protect NE from proteolysis, thus enhancing their activity. Indeed, these exosomes bind and degrade ECM, causing the hallmarks of COPD [94,95].

The proinflammatory role exerted by EVs in COPD has also emerged. Cordazzo et al. carried out an in vitro study in which human mononuclear cells were exposed to CSE to test whether it could induce the release of microparticles [96]. Their data demonstrated that CSE-induced mononuclear cells produced microparticles with a proinflammatory potential. Indeed, their incubation with bronchial epithelial cells induced the expression of proinflammatory mediators such as IL-8 and MCP-1 [96]. Tan et al. investigated the levels of circulating exosomes concerning systemic inflammation in a cohort of subjects encompassing patients with acute exacerbation of COPD, stable COPD patients, and healthy non-smoking individuals [97]. Interestingly, COPD patients showed elevated levels of MVs correlated with the levels of systemic inflammatory markers, such as C-reactive protein (CRP), soluble tumor necrosis factor receptor-1 (sTNFR1), and IL-6 [97,98]. 

Soni et al. analyzed a cohort of 62 patients ranging from mild to severe COPD and identified different MV populations’ biological fluids such as plasma and BAL [99]. They found that BAL neutrophil MVs were the population correlating with some clinical evaluation scores. Their data suggested the potential role of BAL neutrophil MVs as COPD biomarkers, providing a link between the intra-alveolar neutrophil activation (a key mechanism in COPD physiopathology) and clinical outcomes [99]. 

## 8. Extracellular Vesicles and Macrophages in COPD

**Figure 4:**
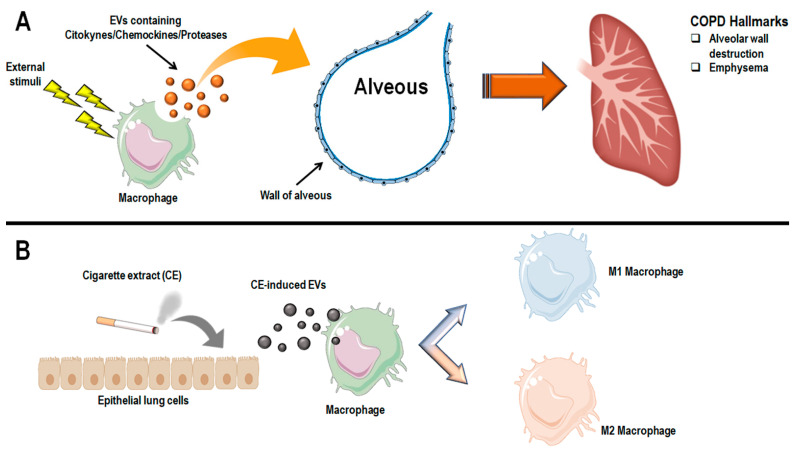
Macrophages are both producers and targets of extracellular vesicles (EVs). (**A**) Macrophage-derived EVs can contain inflammatory effector molecules (e.g., cytokines, chemokines, adhesion molecules, and proteases), which are involved in pathological hallmarks of COPD, such as alveolar wall destruction and emphysema [7,96,100,101]; (**B**) macrophages could also be the target of EVs. Emerging evidence shows that lung cells upon stimulation (e.g., cigarette extract) secrete EVs that target macrophages, influencing their polarization status and function [103,104,105,107].

The increased interest in macrophages and EVs in COPD is mainly due to the speculative hypothesis derived from the pivotal role played by these cells in disease physiopathology and the potential of EVs to influence pathological and physiological conditions. The body of literature sustains that macrophage-derived EVs can contain inflammatory effector molecules, including cytokines, chemokines, adhesion molecules, and proteases, which are involved in pathological hallmarks of COPD, such as alveolar wall destruction and emphysema (Figure 4A) [7,96,100,101]. For example, Li et al. demonstrated that human-cultured macrophages exposed to CSE induced the release of MVs [100]. The authors found that these MVs resulted in proteolytic activity, mainly due to the single transmembrane protease of the MMP family, MMP14. Of note, a 20-h exposure of macrophages to CSE seemed to induce higher levels of MMP14-positive MVs. This confers gelatinolytic and collagenolytic properties, suggesting a role in matrix destruction [100]. Recently, the analysis of the BAL in a cohort of smoker- and non-smoker-COPD patients and healthy non-smoker subjects revealed an increase in the number of AM-derived MVs. In particular, these AM-derived MVs were higher in the BAL of smoker patients suffering COPD and correlated with the degree of airway obstruction. These findings shed new light on the role of these vesicles as mechanistic guides in COPD [102].

Nevertheless, MVs could also exert regulatory activity on macrophages (Figure 4B). In an interesting study, Serban and colleagues used human endothelial cell lines, mouse models, and plasma samples from human individuals to study the effect of endothelial cell releases of MVs induced by CS [103]. Interestingly, the cargo of these MVs was enriched in miRNAs, such as let-7d, miR-191, and miR-126, which induced efferocytosis impairment on recipient macrophages [95,103]. He et al. demonstrated that human bronchial cells derive EVs containing miR-21 target macrophages and influence their polarization toward the M2 phenotype [104]. Recently, Wang et al. studied the effect of CS-induced exosomes released by airway epithelial cells (AECs) on macrophages in both in vitro and in vivo models [105]. Their data revealed that CSE-treated AECs-derived exosomes promoted M1 polarization in both models used. This led the authors to speculate that the exosome-induced M1 polarization might be a possible mechanism by which CS may trigger COPD progression. 

Moreover, they revealed that the exosomes released by AECs following CSE exposure seemed to aggravate pulmonary function impairment due to CS through the upregulation of the triggering receptor expressed on myeloid cells-1 (TREM-1) [105]. Of note, this factor has been demonstrated to induce the polarization of macrophages toward the M1 phenotype [105,106]. Another recent in vitro study argued for the exosome-mediated crosstalk between lung epithelial cells and AMs [107]. The authors used the exosomes derived from CSE-traded Beas-2B cells (human cell line) to investigate their role in macrophage polarization. Interestingly, the exosomes from traded cells induced both M1 and M2 polarization in a dose-effect manner. The secretion of factors, such as TNFα, IL-6, iNOS, IL-10, Arg-1, and TGF-β, was further promoted by these exosomes. Moreover, the authors analyzed the miRNA cargo of the CSE-traded Beas-2B-derived exosomes. They found 27 significantly changed miRNAs in exosomes from CSE-traded cells with respect to control, nine of which were associated with macrophage polarization. Of note, the downregulation of miR-21-3p or miR27b-3p was able to revert the promoting effect of CSE-traded Beas-2B-derived exosomes on macrophage polarization [107].

According to these findings, we could speculate that few data are available describing the macrophage-derived MVs in COPD. Thus, more studies are needed to dissect these cells’ apparently pivotal role in COPD pathogenesis [108]. On the other hand, the paracrine communication between lung epithelial cells and macrophages via EVs may represent an intriguing strategy to improve knowledge about the mechanisms leading to COPD. Of note, EV-containing miRNAs could be suitable targets for developing novel therapeutic and/or diagnostic agents [73]. 

## 9. Conclusions and Future Perspective

COPD is a complex heterogeneous disease, but its pathophysiology is mainly driven by a few major processes, such as airway inflammation, lung tissue damage, and airway remodeling [2,108]. This review focuses on the role of macrophages in COPD. The body of literature suggests that these cells play a significant role in the pathogenesis of the chronic inflammation and aberrant repair evidenced in COPD [44]. Although these cells are pivotal players in the pulmonary protective mechanisms, they could also contribute to lung tissue damage by promoting a dysregulated inflammatory response. This could, in turn, lead to alterations in tissue repair along with a persistent state of chronic low-grade lung inflammation [46]. 

Moreover, oxidative stress and impaired macrophage functions seem to be characteristic in COPD and exacerbations. Oxidative stress and macrophages appear to be strictly interconnected in exacerbations by mutually fueling. Impaired macrophages are an important source of ROS and RNS, and oxidative stress has a profound negative impact on macrophage function and polarization [3]. 

Macrophage plasticity is another key feature in COPD. The alterations in the lung microenvironment may induce different phenotypes in macrophages that result in various functional properties. Differences and changes in macrophage phenotypes are described in lung diseases, including COPD. The body of literature suggests that modifications in interactions occurring among macrophage polarization subsets and changes in their function may profoundly impact disease. Thus, the scientific community seems to agree on this mutualistic vision rather than focusing on a particular dysfunctional phenotype [6]. 

Although the sum of this evidence indicates that macrophage dysfunction plays a central role in COPD, the underlying reasons for these observations need to be further elucidated. In-depth knowledge of the mechanisms behind macrophage anti-inflammatory and reparative functions could contribute to the identification of tailored therapies to counteract COPD outcomes. The commonly used strategies for COPD are based on Bronchodilation, anti-inflammatories, and anti-infection drugs [44]. Although these treatments seem to improve COPD symptoms and prevent exacerbations, little is known about their effectiveness over time on airway remodeling and/or obstructive airflow improvements. Thus, innovative approaches based on changes in behaviors of macrophages could be promising in weakening chronic inflammation and improving the normal repair of lung tissue [44]. For example, macrolides, besides their anti-inflammatory properties, seem to target macrophage key dysfunctional features and, interestingly, increase their ability in efferocytosis. This could be promising for the development of tailored strategies aiming at lowering inflammatory response and increasing tissue repair [44]. Moreover, statins, given their anti-inflammatory and anti-oxidant properties, seem to be intriguing candidates for effective COPD treatments. Of note, these molecules show potential benefit in weakening the chronic inflammatory response mediated by macrophages in COPD [44].

Again, improving a particular macrophage phenotype or an induced change in polarization could provide a novel therapeutic approach for COPD. The use of antioxidant compounds or anti-inflammatory molecules, such as IL-10, as modulators of macrophage plasticity, may offer novel targets for understanding their potential. Moreover, further studies may present more insights regarding the contribution of local microenvironments on macrophage function and polarization status. 

In this scenario, emerging evidence points to EVs as promising candidates in understanding crosstalk and tissue remodeling processes involved in COPD pathogenesis [108]. EV composition and cargo may influence many COPD-related aspects, including macrophage dysfunctions. Macrophages are both producers and targets of MVs (Figure 4). Macrophage-derived EVs can contain inflammatory effector molecules, including cytokines, chemokines, adhesion molecules, and proteases, which are involved in the pathological hallmarks of COPD. On the other hand, MVs seem to perform a critical role in cross-communication between epithelial cells and macrophages. The vesicle cargo produced by lung cells can reach macrophages and influence their functions and polarization status. Overall, this evidence is proof of the promising potential of MVs as powerful packages of cellular information and physiological/pathological influencers through the uptake of their cargo in target cells [75]. Nevertheless, the research of MVs in COPD is still nascent, and more studies are needed to dissect their role and functions further. The use of standardized methods to characterize vesicle isolation, the setup of tailored in vivo studies, and an in-depth characterization of bioactive cargo of these vesicles emerge as pivotal tools to understand their potential in COPD prognosis and management fully. 

## Figures and Tables

**Table 1:** Schematic representation of the macrophage phenotypes and their characteristics (N.A. = not applicable).

Polarization Status	Subset	Inducer	Features	References
** 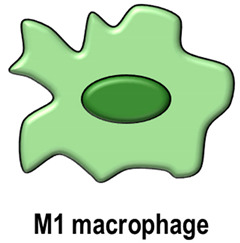 **	N.A.	INF-γ LPS TNF-α	Release of proinflammatory cytokines, such as TNF-α, IL-12, IL-15, IL-23, IL-1β. Production of ROS. Antimicrobial and cytotoxic activities. Immunostimulatory effect.	[6,31,33,34,36,37,38]
** 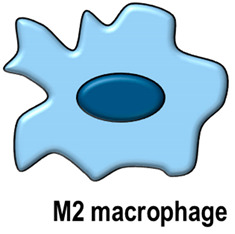 **	M2a	IL-4 IL-13	Negative regulation of pro-inflammatory molecules, such as INF-γ, TNF-α, IL-6, IL-12, IL-1β	[36]
	M2b	immune complex IL-1 receptor ligands LPS	Decrease of IL-12 levels. Increased production of IL-10.	[34,36]
	M2c	glucocorticoids TGF-β or IL-10	Low production of pro-inflammatory cytokines.	[33,36]
	M2d	tumor associated factors	Reduced expression of IL-12. Enhanced release of IL-10.	[36,39]
